# Charged Particle Dynamics in Dry Powder Inhalers

**DOI:** 10.1021/acs.molpharmaceut.5c00485

**Published:** 2025-08-13

**Authors:** Connor Williamson, Joshua Baptiste, Melanie Hamilton, Cheng Pang, David Prime, Anthony J. Stace, Elena Besley

**Affiliations:** † School of Chemistry, 6123 University of Nottingham, University Park, Nottingham NG7 2RD, U.K.; ‡ Drug Product Design Development, GlaxoSmithKline RD, Park Road, Ware, Hertfordshire SG12 0DP, U.K.

**Keywords:** electrostatic interactions, cluster growth, dry powder inhaler, particle dynamics

## Abstract

Mechanisms for the growth of particles in a stream of aerosolised inhalation powders have been investigated computationally using experimentally measured stream compositions. Many-body electrostatic theory has been incorporated into classical particle dynamics simulations to describe the aggregation of charged, fine powder particles in the single and dual stream geometry of an inhaler. The simulations use experimental bipolar charge measurements recorded using a Dekati BOLAR as input. Evidence of a subtle relationship between charge and the dynamics of particle growth contributes to our understanding of the electrostatics and many-body interactions in inhalation powders. It is found that certain combinations of particle size and charge result in a scavenging process whereby small particles, which may be therapeutic, aggregate with large particles to become ineffective due to an overall increase in size. This process may have important implications for design of dry powder inhaler devices.

## Graphical abstract

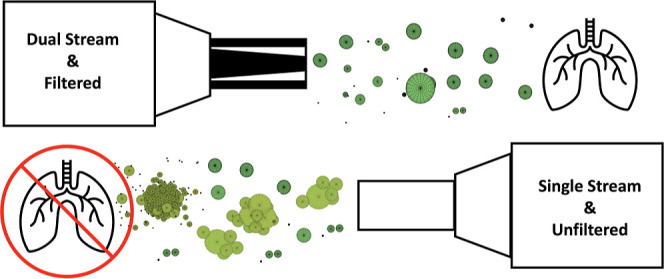

## Introduction

Electrostatic tribocharging of pharmaceutical powders can adversely affect formulation and aerosolisation processes, manufacture and handling procedures, and alter the properties of powder flow.^1–4^ Finding solutions to control the effects caused by particle charging in powder delivery systems, such as metered dose inhaler or dry powder inhaler (DPI), could offer the potential to improve device dosing consistency and targeted deposition in the respiratory system.^5–7^ In pharmaceutical DPIs, active particles, both in-flow and on surfaces, possess a bipolar charge distribution which may influence their aggregation and deposition onto a large permeable membrane of the lungs.^6,8,9^ A number of experimental techniques developed to measure the electrostatic charge of powders^10–14^ are based on the Faraday well, a well-known method for measuring the net charge of bulk powders. However, with regards to DPIs, these measurements are often conducted in an uncontrolled environment, leading to limited reproducibility and insight into the electrostatic characteristics of dispersed aerosols, the dynamics of dose emission, and the effects of agglomeration. In particular, the net charge of the powder does not provide information regarding the bipolar nature of the charge on individual particles or agglomerates which affects their coalescence and, consequently, product performance.

It is currently understood that particles with the aerodynamic diameter of less than 5 μm are most likely to reach and deposit in the pulmonary regions of the lungs, however, according to the literature some particles with the diameter up to 10 μm can still penetrate into the lungs.^15–17^ The aerosolisation process within a DPI is understood to be complex, but it is conventionally viewed as dispersion of a powder dose into the air-stream and further separation of agglomerates, largely due to shear forces, turbulence, and collisions with the walls which occur before the powder has left the device. These dynamic processes may lead to particle deaggregation and reaggregation even if charge–charge interactions are neglected. Computational studies,^18^ for example, included aerodynamic and drag, pressure gradient, lift and gravitational forces to model aggregation, deaggregation, and subsequent reaggregation (with carrier as charge scavenger) phenomena in simulation of high-loading cohesive particles flow inside DPI-like geometries.

The bipolar charge state of particles, following their transmission through a DPI, suggests that aggregation is dominated by Coulomb forces in this case as each released drug dose consists of a wide range of particle sizes and charges. During in-flow collisions, the varied composition of a particle stream has been shown to promote additional attractive interactions at short separations, even between particles of the same sign of charge; the latter process being driven by charge-induced polarization effects.^19^ The attraction between like-charged particles at short separation distances is comprehensively captured by the electrostatic models which include charge-induced polarization effects.^20,21^ As previously shown^22^ the effect of van der Waals forces on the distribution of particle surface polarization charge is negligible, and the van der Waals force overlaps almost entirely with the attractive part of the electrostatic force.

In inelastic collisions, often present in fine powders, like-charged particles with the initial velocity sufficient to overcome the Coulomb barrier, can lose some of the kinetic energy and form stable aggregates. As seen previously,^19,23,24^ attractive interactions between like-charged particles can play a significant role in aggregation, sometimes leading to undesirable growth (“charge scavenging”) prior to deposition. Previous analysis^23,24^ also indicates that collision velocity plays a key role in these inelastic, electrostatically driven aggregation processes.

A Dekati bipolar charge analyzer,^25^ BOLAR, has been used previously to measure the net and bipolar charge distribution of aerosolised lactose (the primary ingredient in many DPIs) as a function of particle size, under the controlled conditions of temperature and humidity.^26^ In this work, the experimental measurements have been incorporated into a computational model of agglomeration under conditions analogous to those present in a DPI, leading to better understanding of the behavior of charged particles in an emitted stream. The many-body electrostatic methods,^19,21,27^ suitable for describing charge-induced interactions including polarization effects, have been combined with classical particle dynamics to simulate fine powder streams traveling through an inhaler or human airway. DPIs are highly diverse in design and operation, and products containing two or more active components (combination products) have proliferated. Some devices separate active components to increase shelf life and facilitate the manufacture of products with multiple variations of active components. These products may have dual rather than single airflow paths within the device. Possible outcomes of particle aggregation in single and dual stream devices have been analyzed, with the role of larger highly charged particles—“potential scavengers” formed from carrier particles—investigated in detail. The nature of the charge scavengers has been described previously.^28–30^ If such particles are present in a stream, they may be able to readsorb smaller pharmaceutical particulates even after the “decoating” precautions.^29^ Numerical simulations of this nature, supported by experimental data, could provide valuable insight into the development of inhalers with improved drug powder flow.

## Materials and Methods

In Dekati BOLAR,^25^ a collection of particles or droplets, e.g. a drug dose or drug analogue, aerosolised at a typical flow rate of 60 L/min is split equally by the flow divider for aerodynamic size differentiation and filtration at the impaction stages (see Figure S1 of Supporting Information). The dose, divided into six fractions, travels beyond the impaction stages and gets separated by the bipolar charge detection tubes. The inner detector (ID) surfaces are charged to +1 kV to attract negatively charged particles, and the outer detector (OD) surfaces are held at ground potential to collect positively charged particles. This allows for the total positive and negative charge of each size fraction to be measured, in addition to the measurements of the total mass collected in each detector.^25^

In this work, lactose-based DPI formulations have been used which are known to exhibit bipolar charge characteristics. Size specific samples were separated into five detector tubes using the effective cutoff diameter of DPI stream particles. The total average charge collected by the detectors, $\overset{®}{q_{d}\;}$ , which corresponds to the total average mass, $\overset{®}{m_{d}\;}$ , of each particle size range, are shown in Figure 1a, and the size distribution of the collected particles can be found in Table 1. The average charge on a particle, ${\mathrm{q̅}}_{p}$ , has been estimated using the values of the overall charge, ${\mathrm{q̅}}_{d}$ , and the number of particles, *n*, collected by each detector (Table 2). These results indicate that the value of ${\mathrm{q̅}}_{p}$ is proportional to the square of the radius of a particle (Figure 1b), thus suggesting that charge resides on the particle’s surface. Here, we model the agglomeration of particles in the stream using a mathematical formalism^21^ which accounts quantitatively for polarization of the surface charge on particles during a collision. The dose-to-dose variability of the total charge was found to be similar across the detector tubes and formulations. A small net positive charge was measured in all detector tubes due to an overall higher charge on the positive particles in comparison to that on the negative particles. The charge distribution similar to those shown in Figure 1a have been reported previously.^25,31^

**Figure 1:**
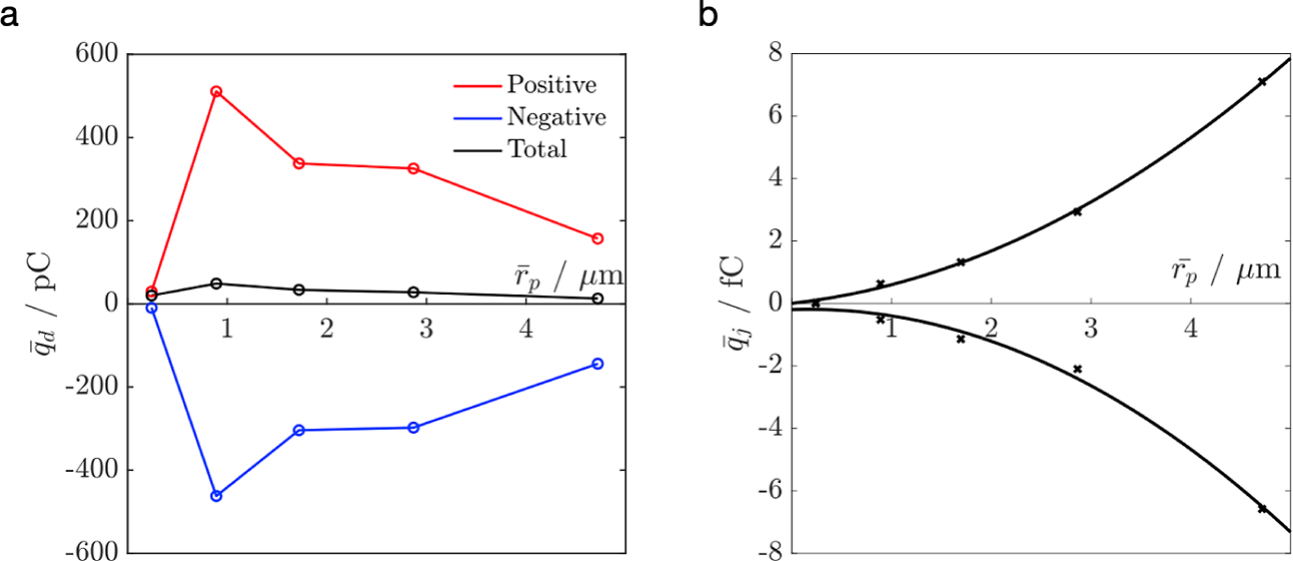
(a) The total average charge, $\overset{®}{q_{d}}$ , measured as a function of the average radius of particles, $\overset{®}{r_{p}}$ , collected separately in each detector tube in the BOLAR experiments; (b) the average charge on a particle, $\overset{®}{q_{p}}$ , as a function of the average radius, $\overset{®}{r_{p}}$ . The magnitude of charge on the positively and negatively charged particles scales as the square of the radius (solid line), with an *R* ^2^ of 0.99.

**Table 1:** Size Distribution and the Average Radius of Particles Collected in Five Detector Tubes of Dekati BOLAR

detector	radius range, μm	average radius, ${\mathrm{r̅}}_{p}$ , μm
1	0.00–0.48	0.24
2	0.48–1.30	0.89
3	1.30–2.13	1.72
4	2.13–3.61	2.87
5	3.61–5.83	4.72

**Table 2:** Mass, Charge, and Distribution of the Particles Collected by the Outer (OD1–OD5) and Inner (ID1–ID5) BOLAR Detectors: ${\mathrm{m̅}}_{d}$ is the Total Average Mass and ${\mathrm{q̅}}_{d}$ is the Total Average Charge Collected by the Detectors^a^

	positively charged particles					negatively charged particles				
detector	OD1	OD2	OD3	OD4	OD5	ID1	ID2	ID3	ID4	ID5
${\mathrm{m̅}}_{d}$ , μg	0.13	3.64	7.93	16.64	14.77	0.14	3.99	8.27	21.23	14.47
${\mathrm{q̅}}_{d}$ , pC	37.60	512.47	334.94	326.66	156.95	–35.91	–465.51	–304.97	–291.93	–142.60
${\mathrm{m̅}}_{p}$ , pg	0.0088	4.49	32.3	151	670	0.0088	4.49	32.3	151	670
*n*, × 10^5^	15.10	8.12	2.45	1.11	0.21	15.40	8.88	2.55	1.41	0.22
*n̅*, %	27.28	14.63	4.41	1.99	0.40	27.74	16.01	4.60	2.54	0.39
${\mathrm{q̅}}_{p}$ , fC	0.02	0.63	1.37	2.95	7.12	–0.006	–0.52	–1.19	–2.07	–6.60

a The average mass of a particle, $\overset{®}{m_{p}}$ , is calculated using the average radius of a particle within a detector ( ${\mathrm{r̅}}_{p}$ taken from Table 1) and the density of lactose of 1.52 g/cm^3^; *n* is the number of particles in a detector, and ${\mathrm{q̅}}_{p}$ is the average particle charge. See Section “Determining the Composition” of Supporting Information for further details.

The total average mass of the collected particles amounted to ${\mathrm{m̅}}_{tot}$ = 91.21 ± 5.91 μg and concentrated predominantly in the detectors OD4 and OD5 (positive particles), and ID4 and ID5 (negative particles). However, the average combined percentage of particles in the detectors OD5 and ID5 is less than 1%, and it is less than 5% in the detectors OD4 and ID4 (Table 2). This suggests that the population of larger particles in the stream is negligibly small. Table 2 also shows that although the detectors OD5 and ID5 contain individual particles of the largest size and the highest charge, the highest total average charge has been collected by the detectors OD2 and ID2. This is due to a significant number of charged particles in the size range of 0.48 to 1.30 μm present in the stream (see Table 2). The experimental results also show that the largest proportion of particles in a DPI stream corresponds to the smallest size, with an average radius of 0.24 μm, which constitute about 27% of both positively and negatively charged streams. Further details on the composition of the expelled DPI streams can be found in Table 2. Particles of excessively large size, such as carrier particles or scavengers typically present in a DPI stream, are collected by the reference filter of the apparatus.

## Results and Discussion

In this work, the many-body solution for the electrostatic interaction energy^21^ has been used in conjunction with Verlet classical particle dynamics (*NVE* ensemble) to study the effect of many-body electrostatic interactions within a DPI stream. A set of differential equations representing the classical equations of motion and the computational setup for this comparatively simple dynamic model has been adopted from ref 19. The use of the full multipolar electrostatic model is essential for a quantitatively accurate estimation of the electrostatic energy (or force) regardless of the specific type of aggregation. Particle polarizability plays a key role in the outcome of individual trajectories.^19^ Where polarizability is absent, particles do not aggregate in low velocity collisions, and collisions can become increasingly destructive as the relative velocity goes up. By contrast, polarizability contributes significantly toward clusters retaining particles following a collision. Polarisability also determines cluster geometries, and the nonadditivity of such interactions has been quantified previously.^27^

A single stream of DPI particles has been modeled as a collection of hard spheres with dimensions and charges described in Table 2, initially moving with a constant velocity. Following an earlier publication,^32^ the particles were assigned a coefficient of restitution of 0.8 to allow for the energy loss through inelastic collisions. No interactions with the walls were considered as the DPI stream was assumed to have much narrower dispersion than the dimensions of human airways. In the stream, charged particles in a close proximity cause redistribution of surface charge due to distance dependent many-body electrostatic interactions, which can be described using multipolar expansions of spherical harmonics.^19,21,24^ Using a fast multipole method, Hassan et al.^21^ describe these interactions accurately at a linearly scaling computational cost with respect to the number of particles, and the solution yields the total electrostatic interaction energy, the surface charge distribution, and the force acting on each particle. Lindgren et al.^19^ show that it is possible to combine such a solution with classical particle dynamics simulations to study the time evolution of many body collisions. Further details are discussed in Section “Modeling Electrostatic Interactions of Polarisable Particles” of the Supporting Information.

All considered interactions are assumed to occur in vacuum (ϵ = 1). While acceptable for modeling electrostatics, this assumption neglects drag forces which might not be negligible in a real DPI where drag forces may influence particle aggregation and dispersion, impacting drug delivery to the lungs. These forces arise from the airflow generated during inhalation and can affect how drug particles move within the inhaler and ultimately to the lungs. Several factors, such as airflow rate, particle size and shape and inhaler geometry, may affect the contribution from drag forces.^33^

We first consider single collision events taking place in a stream, which had previously been shown to affect particle coalescence in air; for example, in the agglomeration of charged ice and dust particles in the mesosphere and lower thermosphere.^23^ In the collision scenarios considered in this work, a pair of particles form a cluster only if they carry opposite charge as any like-charge aggregation as first described in^20^ is negligible. As our experimental measurements show (Table 2), the majority of particles emerging from a DPI are collected in the OD1 and ID1 detectors, therefore clusters containing pairs of the smallest particles ( ${\mathrm{r̅}}_{p}<$ 0.48 μm) are expected to be common. The collision dynamics in a DPI stream between a small pair and a large carrier particle is described in Figure 2 which highlights a potential scavenging mechanism^29^ that could occur in the stream, given the flow rate of 60 L/min and the diameter of the device outlet of 1.4 cm.

**Figure 2:**
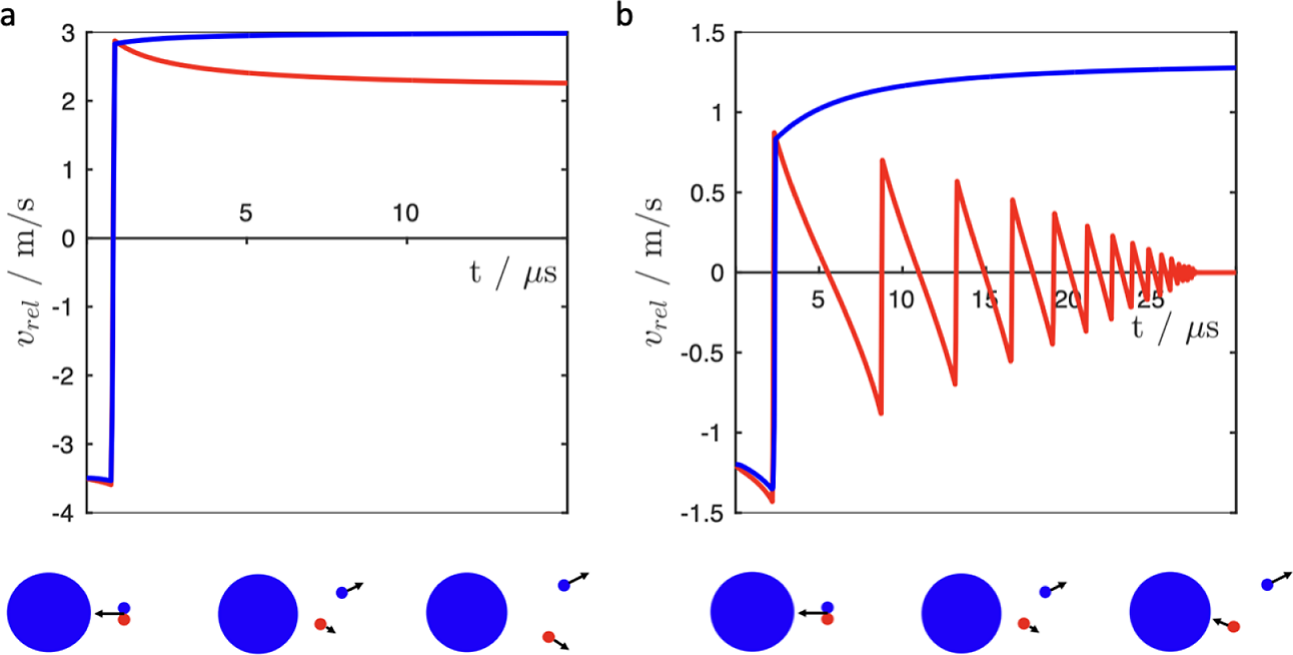
Relative velocity, *v*_rel_, of two small oppositely charged particles, positive (red) and negative (blue), in a collision with a large negatively charged stationary target as a function of time: (a) initial *v*_rel_ = 3.5 m/s, (b) initial *v*_rel_ = 1.2 m/s. The smallest size fraction collected by the ID1/OD1 detectors is used (*r*_p_ = 0.24 μm, *q*_+_ = 0.02 fC, *q*_–_ = −0.006 fC), and the large particle is taken from the ID5 detector (*r*_p_ = 4.72 μm, *q* = −6.60 fC).

In the simulations shown in Figure 2, the large negatively charged particle is stationary and a pair of oppositely charged small particles is initially moving toward the large target, as depicted by the velocity vectors. The initial surface-to-surface separation distance between the target and the pair is 3 μm, and the initial incoming relative velocity of a small pair plays a critical role in the dynamic coalescence. Collisions shown in Figure 2 typically destroy the small cluster, but if the initial velocity of the pair is relatively low, these collisions also provide a route to the formation of a larger bipolar particulate. In Figure 2b, the small positively charged particle loses its kinetic energy through a number of consecutive collisions with the surface of the large negative particle, and it eventually gets firmly attached to the surface while the negatively charged small particle breaks away. This exchange step could be critical in cluster formation in a DPI stream, making the product of the collision—a pair of oppositely charged particles with drastically different sizes—even more populous than the original small pair.

A pathway to the formation of triplets and quartets (clusters containing three or four particles, respectively) is shown in Figure 3. Two small negative particles 1 and 2 are stabilized initially on the surface of the large positively charged (lactose) carrier through the dissipation of the kinetic energy in the first few microseconds of a simulation (inset in Figure 3b,c). The residual velocity at times shorter than 5 μs indicates a slow transition into a more stable configuration where the negative particles 1 and 2 move further apart. This relaxation process is much slower than the movement of the incoming particle 3 which has the initial relative velocity of 0.3 m/s. As particle 3 gets closer to the lactose carrier it accelerates toward it driven by Coulomb attraction. After the collision, particle 3 is also stabilized on the surface leading to further cluster growth. The incoming trajectory of particle 3 represents the most repulsive case as the other two negative particles attached to the surface of the carrier face directly the approaching negative particle 3. This collision has also resulted in coalescence. Given the correct conditions, this cluster growth process, known as charge scavenging, will persist in a DPI stream.

**Figure 3:**
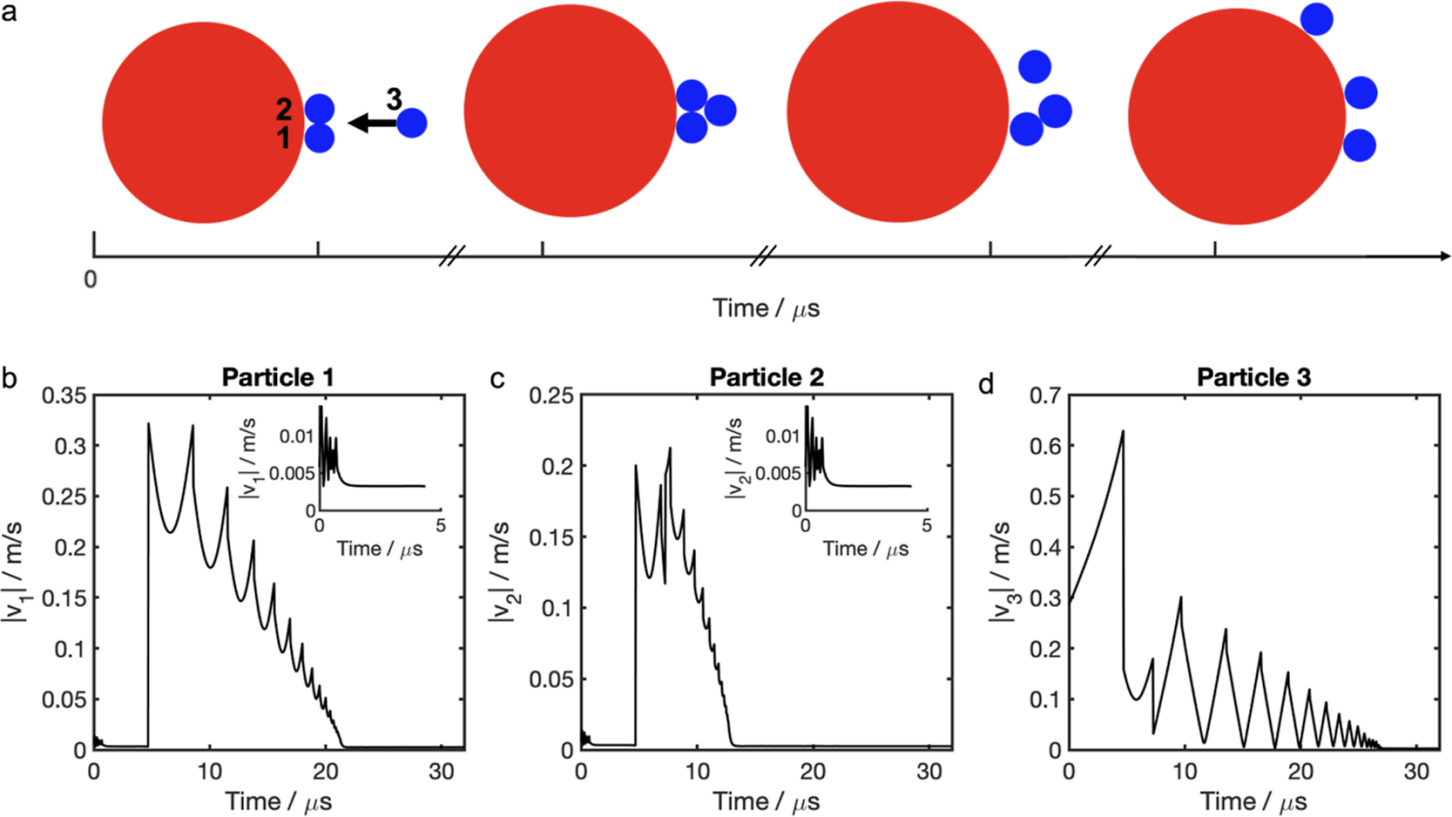
Initial steps in scavenging of small charged particles (*r*_p_ = 0.24 μm, *q* = −0.006 fC) by a large (lactose) carrier (*r*_p_ = 4.72 μm, *q* = 7.10 fC): (a) snapshots of the particle dynamics simulation at 2 μs, 8 μs, 12.5 μs, and 20 μs, (b–d) velocity of the smaller particles during the scavenging process. Particle 3 has initial relative velocity of 0.3 m/s. The insets in (b–c) depict the velocity for the first 5 μs s of the simulation.

Particles of larger size occasionally enter a DPI stream. Initially, these large carriers (or charge scavengers) aid the smaller active pharmaceutical ingredients (API) in gaining the velocity and direction required for inhalation and release into the stream. Subsequently, they are often ejected through collisions with the walls before entering the mouth or trachea;^29^ however, given the size and charge of the scavengers,^6,15^ further interactions between particles in the stream could lead to readsorption of the API onto the scavenger and prevent deposition into the lungs. To understand the mechanisms leading to readsorption of the API within a DPI stream, a more complete particle dynamics simulation of the stream containing potential charge scavengers has been undertaken.

In this computational setup, a charge scavenger travels through a DPI cloud consisting of 300 particles each with a dielectric constant of 2.9 (100 negatively and 100 positively charged particles with *r*_p_ = 0.24 μm, 40 negatively and 40 positively charged particles with *r*_p_ = 0.89 μm, and 10 negatively and 10 positively charged particles with *r*_p_ = 1.72 μm). The stream velocity used in the simulations corresponds to a DPI flow of 60 L/min (0.001 m^3^/s) through a circular outlet with a diameter of 1.4 cm (the corresponding area is 1.54 × 10^–4^ m^2^). In the absence of drag forces in these simulations, the carrier charge scavenger particle is therefore assumed to have the initial velocity of 6.5 m/s. The charge on the particles is taken from Table 2. The size of the scavenger (*r*_p_ = 10 μm) corresponds to one of the smaller carrier particles found within a commercial DPI stream.^15^ The particle cloud has been generated by randomly placing the particles within a box with dimensions of 73.1 μm at the center of the simulation cell which is ten times bigger. This eliminates the effect of collisions with the walls. The simulation time has been increased to 200 μs. All interactions are assumed to take place in a vacuum (dielectric constant of 1) at room temperature. Random initial velocities are assigned to the particles in the cloud following the Maxwell–Boltzmann distribution determined by the collisions.

Figure 4 shows that if a large scavenger is present within a stream, spontaneous aggregation of smaller API particles on its surface occurs readily, thus preventing the active API from reaching the lung membrane. For a negatively charged scavenger (*q* = −38.5 fC, in this case), all particles that aggregate onto the surface have positive charges. The majority of particles aggregated on the scavenger are of the smallest size fraction (Figure 4a), with the percentage uptake of particles with the radius of 0.24 and 0.89 μm being very similar. The aggregation rate is mainly determined by the change in the total charge of the scavenger (Figure 4b) as the added mass makes a very small contribution to its size. On average, 30% of smaller particles (radius of 0.24 and 0.89 μm) have been adsorbed onto the scavenger, yet the aggregation of larger particles (radius of 1.72 μm) is only about 5%.

**Figure 4:**
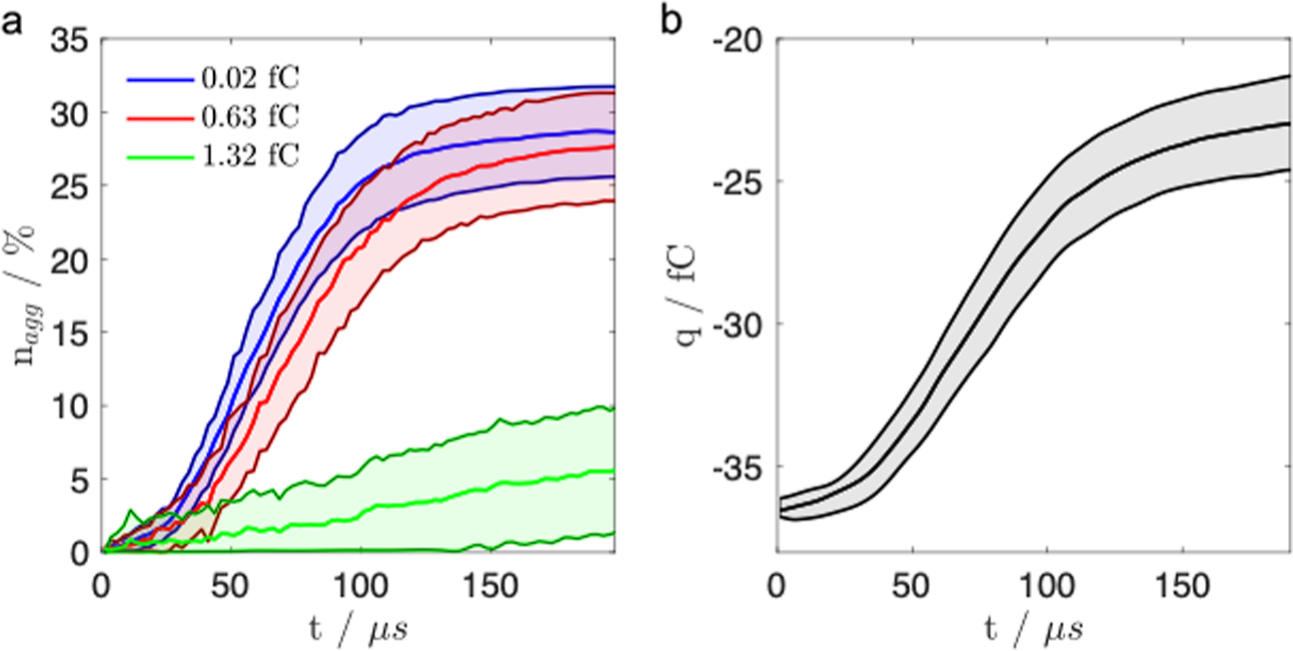
Aggregation outcomes for a scavenger (*r*_p_ = 10 μm, *q* = −38.5 fC) passing through a cloud containing 300 particles: 200 particles with *r*_p_ = 0.24 μm (blue), 80 particles with *r*_p_ = 0.89 μm (red) and 20 particles with *r*_p_ = 1.72 μm (green): (a) the percentage of particles in a cluster, (b) the net charge on the scavenger postaggregation. The number of positive and negative particles in the cloud is equal. The shaded regions indicate the standard error of the obtained results.

Further analysis (see Section “Charge Scavengers” of the Supporting Information) shows that if the charge on the scavenger is halved (*q* = −19.3 fC) half the amount of aggregation occurs within a stream. This decrease is due to a 2.5 times lower aggregation rate as compared to the case shown in Figure 4. The simulations also reveal that the aggregation outcome for a positively charged scavenger with *q* = 46.3 fC is halved in comparison to the case shown in Figure 4 for the negative scavenger with *q* = −38.5 fC. This can be attributed to the uneven bipolar nature of the stream (see Table 2) so that positively charged particles that aggregate onto the negative scavenger (Figure 4) carry 3.5 times more charge than their negative counterparts. In larger streams, the shape of the resultant agglomerates remains predominantly spherical (Figure 5a) as the scavenger continues to adsorb particles to its surface in size order—from smallest to largest—as seen by the gradients of the curves in Figure 4. Other smaller assemblies are also formed, two of which are shown in Figure 5b,c. These clusters could also become too large to be adsorbed into the lungs, and their formation in the stream should be minimized.

**Figure 5:**
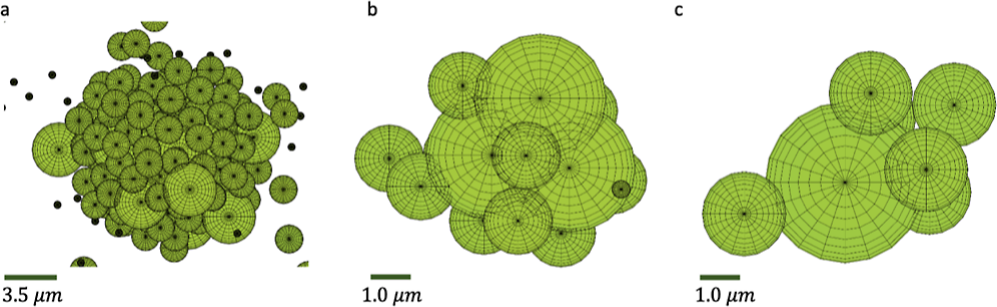
Examples of large clusters formed in a single simulation of a DPI stream containing a charge scavenger and 1500 smaller particles 500 negative and 500 positive particles with *r*_p_ = 0.24 μm, 200 negative and 200 positive particles with *r*_p_ = 0.89 μm, and 50 negative and 50 positive particles with *r*_p_ = 1.72 μm. The simulation time is 200 μs.

In conclusion, even for the smallest carrier particle used in dry powder inhalers (*r*_p_ = 10 μm), charge scavenging may cause readsorption of API particulates and reduce the required amount of the API during inhalation leading to the use of higher doses. This effect will be even more pronounced for the larger carriers. As charge scavenging has been shown to remove more than a quarter of the stream particles of smaller size fractions, the elimination of charge scavengers from the DPI stream before entering the mouth/trachea should be considered. Additionally, reducing the rate of higher energy collisions between particles would help to avoid the formation of larger clusters shown in Figure 5.

In order to prevent aggregates from becoming too large to permeate the lung membrane, one may consider altering the stream configuration to reduce the high energy collisions and charge scavenging and to manipulate the size range of the particles in the stream. Multiple propulsions of less dense streams could also reduce the number of collisions and hence clustering. This can be realized in two adjacent DPI streams composed of particles of the smallest three size fractions and directed toward the target at a small angle. A simple schematic of such design can be found in Section “Dual Stream Design” of the Supporting Information.

Dual stream dynamics has been investigated for streams of particles represented by the three smallest sizes. The two streams were directed at a target at a 6° angle with the velocity of 6 m/s. An additional velocity was randomly assigned to each particle to account for thermal fluctuations. These simulations were carried out under the same conditions as previously described. In the dual stream, particle growth appears to be significantly reduced, as shown in Figure 6. The majority of particles do not aggregate, with only about 6% of particles forming clusters after 100 μs of simulation time. This represents a significant reduction in particle aggregation compared to 25–30% aggregation in the case of a single stream (Figure 4). Particles that do form clusters in the dual stream are typically organized in pairs, with some small presence of triplets and even fewer instances of clusters containing four particles. The aggregated pairs frequently feature in the attachment between a larger particle and a smaller particle (Figure 6b).

**Figure 6:**
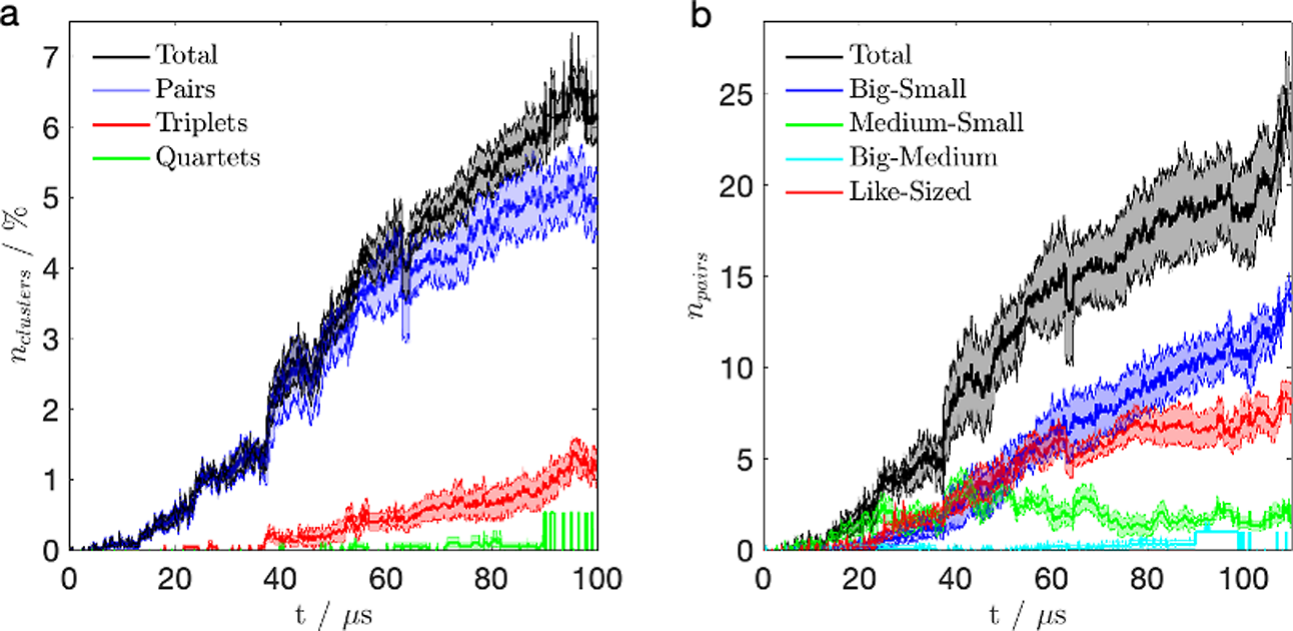
Composition of the dual stream averaged over 10 runs, initially containing 125 negative and 125 positive particles with *r*_p_ = 0.24 μm, 50 negative and 50 positive particles with *r*_p_ = 0.89 μm, and 13 negative and 13 positive particles with *r*_p_ = 1.72 μm: (a) the percentage of clusters in the dual stream showing pairs (blue), triplets (red), quartets (green), and the total percentage of clusters (black); (b) composition of the aggregated pairs including big (*r*_p_ = 1.72 μm)—small (*r*_p_ = 0.24 μm) pair (blue), medium (*r*_p_ = 0.89 μm)—small (*r*_p_ = 0.24 μm) pair (green), big (*r*_p_ = 1.72 μm)—medium (*r*_p_ = 0.89 μm) pair (cyan), and same size particles (red). The total number of pairs is shown in black. The two streams are directed at the target at 6° angle. The shaded regions indicate the standard error of the obtained results.

A small contribution from surface polarization effects, fully accounted in the computational setup as shown in Section “Like-Charge Attraction” of the Supporting Information, is expected to increase the stability of the pairs containing dissimilar size particles compared to like-size configurations.^20–24^ All pairs were found to contain oppositely charged particles, as expected, given the bipolar nature of the stream and low polarizability of the constituent particles. Triplets formed in the stream are composed of one larger particle and two smaller particles with charge opposite to that of the larger particle, similar to the initial geometry shown in Figure 3. The rarely occurring quartets are formed following the mechanism described in Figure 3. This shows that although charge scavenging is limited in the dual stream, it still occurs. Given that the majority of particles emerging from an API are very small, the conclusions cited in ref 15 would suggest their aggregation into larger units could be beneficial, providing that the final particle remains smaller than 10 μm in diameter.

## Conclusions

In summary, our simulations have shown that using two or more streams aimed at a common target and filtering out charge scavengers could enable more efficient drug delivery for the users of DPIs. Within a single stream, a charge scavenger can readsorb up to 25–30% of API particles, hence reducing the API dose reaching the lungs. An inhaler designed with more than one stream and a smaller size range of particles has been shown to limit aggregation over a similar time period. Aerosolisation in DPIs has been shown to be a dynamic process of particle deaggregation and reaggregation. The development of efficient DPI products has historically focused on maximizing deaggregation. This study has shown the importance of also minimizing reaggregation.

## Supplementary Material



## Notes

Experimental data, population analysis, convergence and stability calculations, and the angular dependence of the particle collisions (PDF)

## §

Independent Inhalation Consultants formerly affiliated with Drug Product Design Development, GlaxoSmithKline RD, Park Road, Ware, Hertfordshire SG12 0DP, U.K
