# Supplementary material for: Charged Particle Dynamics in Dry Powder Inhalers
Source: Mol Pharm. 2025 Aug 13;22(9):5485–92. doi: 10.1021/acs.molpharmaceut.5c00485 (PMC12406246; doi:10.1021/acs.molpharmaceut.5c00485)
Supplement: Supplementary file 1 [file mp5c00485_si_001.pdf]

Charged particle dynamics in dry powder inhalers: Supplementary Information

Connor Williamson,[†] Joshua Baptiste,[†] Melanie Hamilton,[‡] Cheng Pang,[¶] David Prime,[¶] Anthony J Stace,[†] and Elena Besley*[,†]

†*School of Chemistry, University of Nottingham, Nottingham, NG72RD, UK*

‡*Drug Product Design Development, GlaxoSmithKline RD, Park Road, Ware, Hertfordshire, SG120DP, UK*

¶*Independent Inhalation Consultant, formerly of ‡*

E-mail: elena.besley@nottingham.ac.uk

BOLAR

As described in the main text, a Dekati BOLAR™ analyses the constituents of an aerosolised flow by segregating particles by their size, and measuring the total mass and charge collected of each of five particle sizes. A schematic of such a device is shown in Figure S1. The flow is initially split into five equal streams, which travel to five individual detectors; each detector is fitted with an impaction stage that limits which particles are allowed through to the detector, depending on their size. Detector 5 allows particles with the largest average radius through, and detector 1 allows particles of the smallest radius through as described in Table 1 of the main text. As each size of particle could be either negatively or positively charged, the particles are separated by sign of charge via the inner and outer detector of each compartment by virtue of the potential at which the OD and ID are held at.

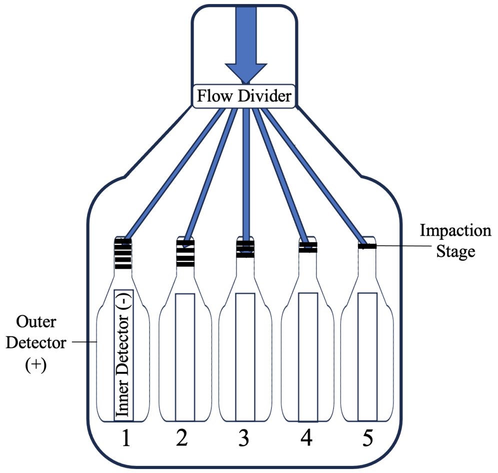

Figure S1: Schematic of Dekati BOLAR™.

Determining the Composition

Given the experimental data provided alongside this supplementary information, it can be seen that it is possible to determine the number of each particle size within a stream, and therefore the average charge per particle. This provides critical insight to the environment of each particle type in the stream. The average radius of each size fraction is assumed to be possessed by all particles within that stream, and, as in the main text, each particle is assumed to be spherical in nature.

Given that the minority of the mass is collected in OD/ID 1, a minority of the collected samples were not large enough to register a mass measurement in ID/OD 1. However, in both cases at least one mass measurement was collected with non-zero mass, whilst all measurements provided a measure of the total charge (both positive and negative). As such, the average mass of the collected particles in OD/ID 1 include runs of zero mass whilst the average charge of both the positive and negative particles includes only runs in which the number of particles can be determined (i.e. those with some mass collected).

Modelling Electrostatic Interactions of Polarisable Particles

Convergence

The many-body framework[1] requires the definition of the degree (number) of functions (spherical harmonics) used to model the surface of each particle undergoing polarisation, such that an infinite number of functions would give an exact answer. The interaction energy of the system can be used to measure the convergence with respect to the number of functions defining each particles surface. Hence, one can determine the number of functions required to model each particles surface.

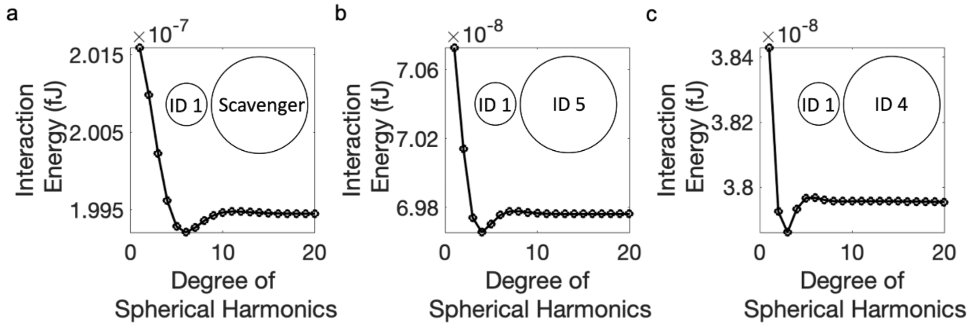

Figure S2: The interaction energy as a function of the number of spherical harmonics used to model the particles surface at approximately 0 separation between a particle from ID 1 (a = 0.24 μm, q = -0.006 fC) and; a charge scavenger (a = 10μm, q = -38.5 fC) (a), a particle from ID 5 (q = -6.5 fC, a = 4.72 μm) (b), and a particle from ID 4 (q = -2.1 fC, a = 2.81 μm) (c).

Through careful analysis of Figure S2, the required number of spherical harmonics can be determined to accurately model the surface of the particles in the system; this is the minimum number required to cause negligible change to the interaction energy. In the case of Figure S2 this is 15, whereas in the case of Figures S2b and c, 13 and 10. The amount of polarisation scales (here) with the size difference of the particles as they carry a similar charge density and are made of the same material. As such, the number of terms required for convergence of the largest particles with the smallest particles is taken as a benchmark.

Like-Charge Attraction

Like-charge attraction, a force caused by polarisation of differently sized particles at close separation, is discussed in the text and throughout the literature.[2–4] However, the tendency to undergo like charge attraction is dependent on the polarisability, charge, size and surface charge of the particles interacting.[1] We show that, for the particles detected in the Dekati BOLAR, no like charge attraction can occur, even in the most extreme of cases. This can be seen in Figure S3.

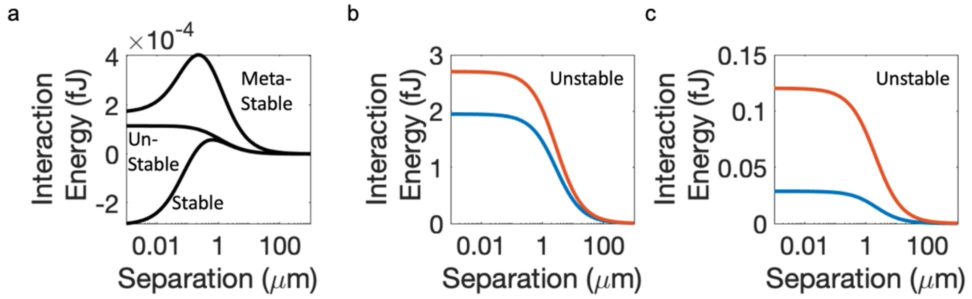

Figure S3: The interaction energy as a function of surface-to-surface separation of two spherical particles. The interaction energies are calculated in (a) are; (Stable) $a_1 = 0.1$ μm, a_2 =1 μm, $q_1 = 70$ fC and q_2 =20 fC; (Metastable) $a_1 = 0.1$ μm, a_2 =1 μm, $q_1 = 70$ fC and q_2 =100 fC; (Unstable) $a_1 = 0.1$ μm, a_2 =1 μm, $q_1 = 70$ fC and q_2 =150 fC. The interaction energy as a function of surface-to-separation between like charged particles detected by the Dekati BOLAR are illustrated in (b) for the case of a particle taken from the detector ID2 interacting with a particle from the ID3 detector (blue), and a particle from the OD2 detector interacting with a particle from the OD3 detector; (c) shows the interaction energy profiles for the pairs from the ID1 and ID3 detectors (blue) and the OD1 and OD3 detectors (red).

Collision Dynamics

The interaction energy ($E_{\mathrm{electrostatic}}$) at the point of contact is calculated using Hassan *et al* solution[1] for each combination of oppositely charged particles and shown in Table SS1. These are the values used as the baseline for the determination of the outcome of a collision between two particles, with the relative magnitude of the kinetic energy of a collision (E_{IK}) compared to the magnitude of $E_{\mathrm{electrostatic}}$ to determine the outcome of the collision.

Table S1: A table showing $E_{\mathrm{electrostatic}}$ at the point of contact in eV for all possible combinations of oppositely charged particles.

	OD1	OD2	OD3	OD4	OD5
ID1	-16	-224	-244	-325	-481
ID2	-563	-11198	-16079	-24367	-38792
ID3	-693	-16837	-26857	-44193	-74946
ID4	-786	-21411	-36975	-65543	-118872
ID5	-1528	-43619	-80827	-153836	-299633

A plot of E_{IK} as a function of the angle of approach of two colliding particles (θ) for the two most asymmetrically-sized particles with radii of 0.24 and 4.72 µm and $C_{\mathrm{R}} = 0.8$, travelling with linear velocities $v_j = 6$ m s^{-1} is shown below in Figure S4. The plot displays a value for E_{IK} for a head on collision ($\theta = 90°$) of around 25 keV which is around 20-50 times the magnitude of $E_{\mathrm{electrostatic}}$ for the relevant interactions shown in Table S1. This is a clear example of a collision with too much energy that would lead to a complete separation of the particles, even given their opposite charge. A closer look at the plot, given the inset in Figure S4, shows the regions where E_{IK} is more comparable to $E_{\mathrm{electrostatic}}$. This region clearly shows points of intersection between E_{IK} and $E_{\mathrm{electrostatic}}$ that signify the critical angles (θ_{crit}) where where any deviation from this angle will alter the outcome of the collision for two particles with this speed. For the interaction between particles from the OD1 and

ID5 detectors (red plot, $E_{\mathsf{electrostatic}} = -1528$ eV), θ_{crit} is around 15°, whereas for the weaker interaction for particles from OD5 and ID1 (blue plot, $E_{\mathsf{electrostatic}} = -482$ eV) has a much lower value for θ_{crit} of 8°.

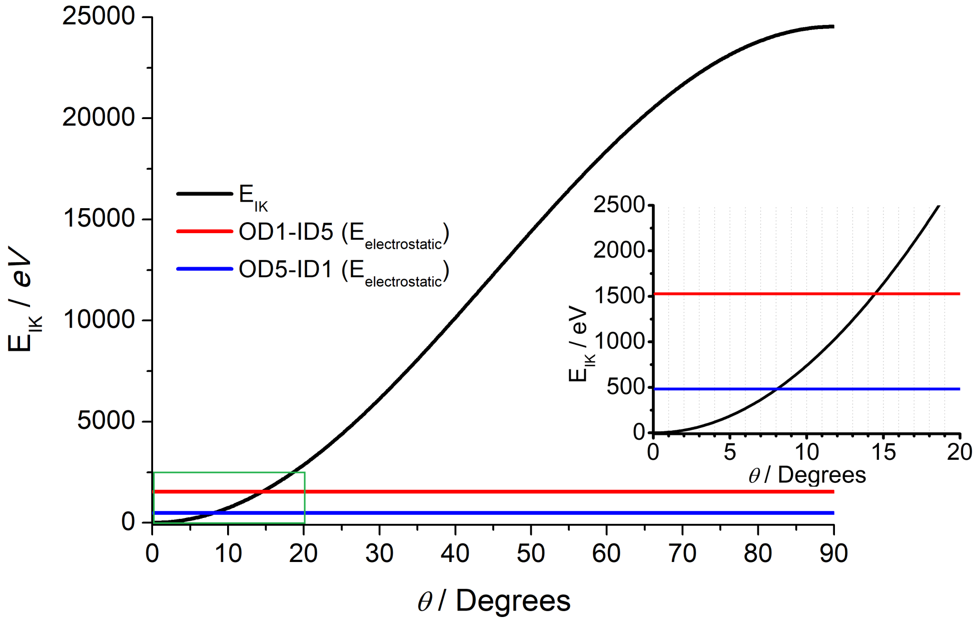

Figure S4: A plot showing the internal kinetic energy of the system E_{IK} as a function of θ for the collision between the largest ($a_1 = 4.72$ µm) and smallest ($a_2 = 0.24$ µm) particles with $v_j = 6$ m s^{-1} (black line). Also shown is $E_{\mathsf{electrostatic}}$ for the interactions between particles where $q_1 = -6575$ aC and $q_2 = +20.24$ aC (red) and $q_1 = +7097$ aC and $q_2 = -5.85$ aC (blue). The inset data shows the region where the 3 plots are comparable in magnitude, indicated by the green rectangle on the main plot.

The contour plots in Figure S5 show E_{IK} for a range of colliding systems, enabling the determination of the outcome of a collision. In each of the figures, the collisions resulting in a value of E_{IK} lower than $E_{\mathrm{electrostatic}}$ are coloured yellow or red, whereas those above this value are in the blue region. The interactions between similarly sized particles are shown in Figures S5a and S5d; these interactions show very small critical angles even at lower velocities v_j of 5 m/s ($\theta_{\mathrm{crit}} < 3°$), indicating a very low probability of particles aggregating in the event of a collision.

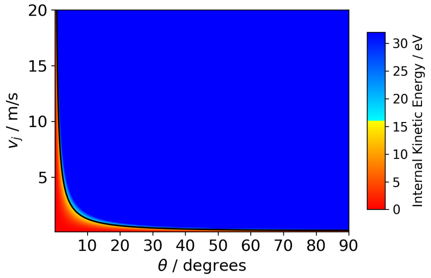

(a) $a_{1,2} = 0.24$ µm, $q_1 = +20.24$ aC, $q_2 = -5.85$ aC

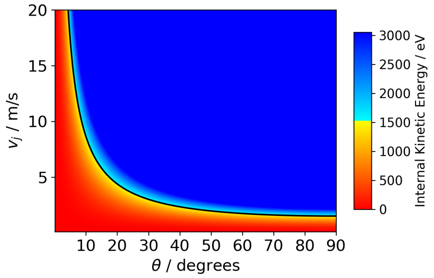

(b) $a_1 = 0.24$ µm, $a_2 = 4.72$ µm, $q_1 = +20.24$ aC, $q_2 = -6575$ aC

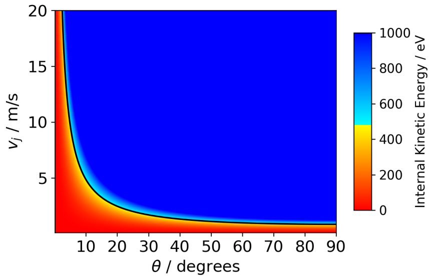

(c) $a_1 = 4.72$ µm, $a_2 = 0.24$ µm, $q_1 = +7097$ aC, $q_2 = -5.85$ aC

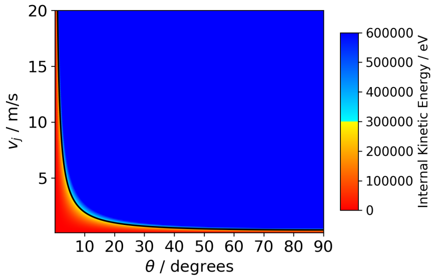

(d) $a_{1,2} = 4.72$ µm, $q_1 = +7097$ aC, $q_2 = -6575$ aC

Figure S5: 2D plots of E_{IK} as a function of v_j and θ for 4 different colliding systems. The interactions presented are those between the smallest (a), most asymmetric (b) and (c), and largest (d) lactose particles. For each interaction, $E_{\text{electrostatic}}$ is represented for each plot as the black contour line, cohesive collisions are indicated by the red/yellow region, whereas the blue region indicates separation.

In contrast, the interactions between asymmetrically sized particles, presented in Figures S5b and S5c, show much larger values of θ_{crit} with fairly significant values ($\theta_{\mathsf{crit}} \geq 5°$) even at larger values of v_j up to 20 m/s. This observation supports the idea that larger charged particles will be more likely to scavenge a collection of smaller particles across its surface after a series of collisions, and are less selective in their aggregation that symmetrically sized particles. As the dielectric properties of the APIs used in the inhalers are comparable with those of lactose, it can be assumed that API/Lactose containing mixtures will behave in similar ways. The particle sizes for each of the APIs are generally smaller than those of lactose implying that, after a series of collisions within the inhaler's manifold, smaller API

particles will typically be deposited across the surface of larger lactose particles.

The coefficient of restitution is defined as the ratio of a particles velocity after a collision to the velocity before the collision. Analysis of both the critical angles θ_{crit} and critical speeds v_{crit} for constant v_j and θ respectively for two systems that are identical with the exception of their values for C_{R} are shown in Figure S6, with the plots shown in Figures S6a and S6b showing the results for C_{R} values of 0.8 and 0.5 respectively.

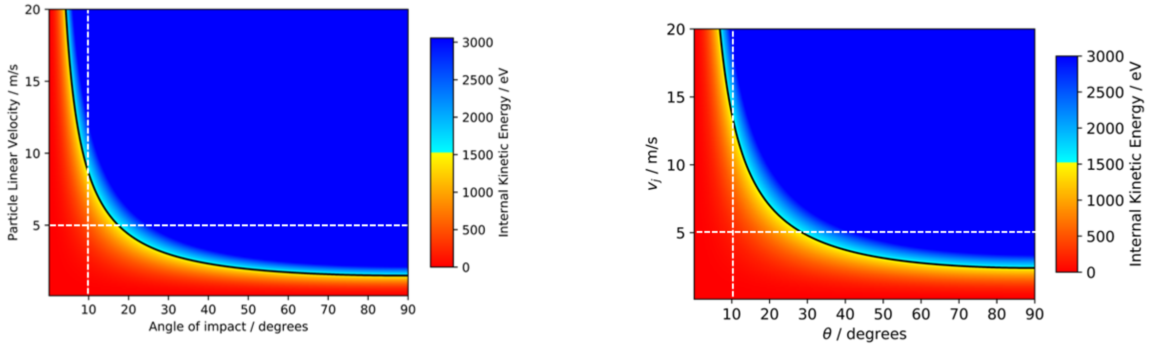

(a) $C_{\text{R}} = 0.8$ (b) $C_{\text{R}} = 0.5$

Figure S6: A comparison of the 2D plots for 2 systems containing a pair of colliding asymmetrically-sized lactose particles ($a_1 = 4.72$ μm, $a_2 = 0.24$ μm, $q_1 = +7097$ aC, $q_2 = -5.85$ aC). The systems are identical with the exception of the coefficient of restitution C_{R}. The white horizontal and vertical dashed lines are placed in identical positions in each figure for constant v_j and θ respectively.

At a first glance the two plots look extremely similar, but a closer look at the plots show a clear difference in the points of intersection between the dashed lines and the black contour line for $E_{\text{electrostatic}}$. The horizontal dashed lines in Figure S6 represent E_{IK} for when $v_j = 5$ m s^{-1}, where the intersection point with the black line indicates the value of θ_{crit} in each case; when $C_{\text{R}} = 0.8$, $\theta_{\text{crit}} \approx 18°$, whereas when C_{R} is given a lower value of 0.5, θ_{crit} increases to $\sim 30°$. A similar pattern is observed with the vertical dashed lines at $\theta = 10°$, revealing critical velocities of ~ 8 m s^{-1} and ~ 13 m s^{-1} for C_{R} values equal to 0.8 and 0.5 respectively.

Charge Scavengers

Charge scavengers are rare, larger, and more highly charged (with a similar surface charge density) particles[5,6] that act as carrier particles. In the manuscript, we use the values for the charge and radius of the charge scavengers as introduced in the main text, with the exception of scavenger particle II that was added to further understand the effect of charge carried by the scavenger.

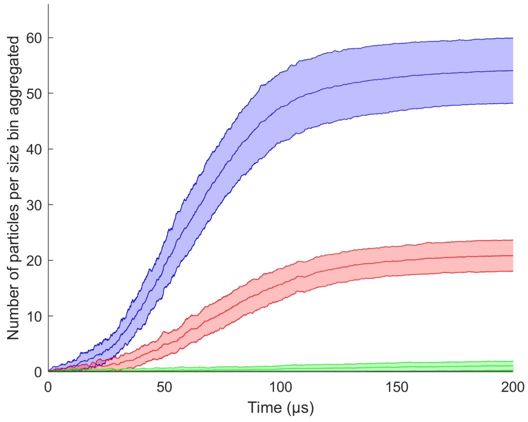

(a)

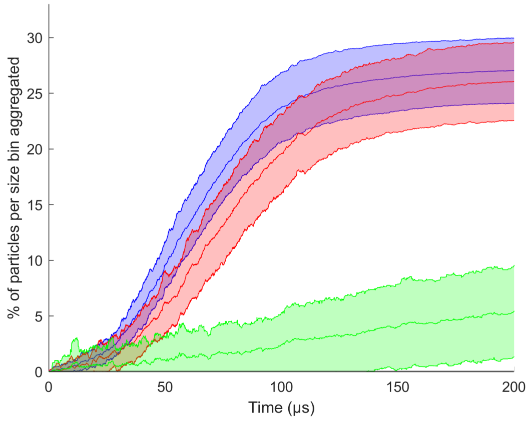

(b)

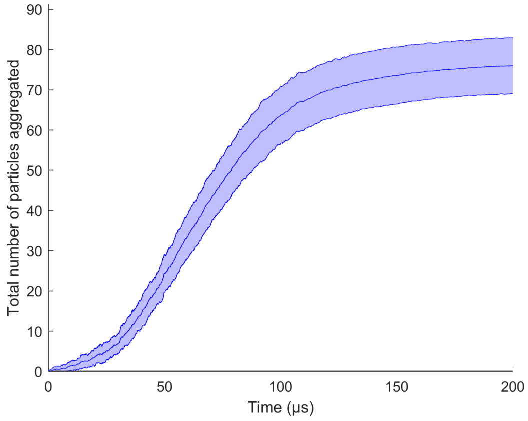

(c)

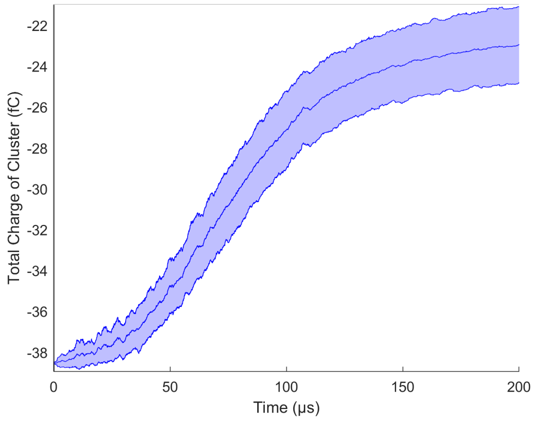

(d)

Figure S7: Aggregation data over 200 μs simulation time for the scavenging particle I ($a = 10$ μm, $q = -38.5$ fC) passing through a cloud of 300 aerosol particles. The number of particles (a) and the percentage of particles (b) coalesced on the scavenger are shown in blue for $r_p = 0.24$ μm, red for $r_p = 0.89$ μm and green for $r_p = 1.70$ μm; (c) gives the total number of particles in a cluster and (d) shows the total cluster charge. Each plot shows the mean and standard deviation for the sample size.

As per the results in the main text, each charge scavenger was simulated passing through a stream of particles, analogous to those detected experimentally, for 200 μs. Whilst the composition and the total charge of particles' aggregation are explored in the main text, the total size and aggregation rate of the cluster is also of interest considering that streams composition and size often place limitations on the size of the formed clusters. This is explored in Figure S7. Figure S8 represents the effect of the bipolar nature of the stream.

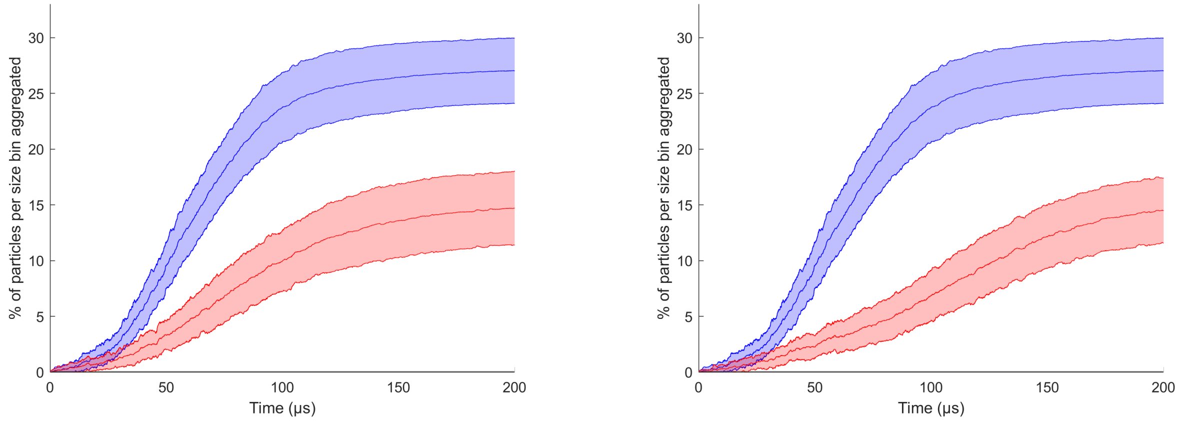

(a) (b)

Figure S8: Aggregation data over 200 μs simulation time for the smallest particles with $r_p = 0.24$ μm attached to scavenger particles with the radius of $a = 10$ μm but with a different value of charge: (a) scavenger particle I has the charge of $q = -38.5$ fC (blue), scavenger particle II has $q = -19.3$ fC (red), (b) scavenger particle I has $q = -38.5$ fC (blue) and scavenger particle III has $q = +46.3$ fC (red). Each plot shows the mean and standard deviation for the sample size.

Dual Stream Design

As presented in the main text, a filtered dual stream inhaler design, in which two (or more) streams are aimed at a common target either before or at the trachea, appears to lead to less aggregation and removes the potential of charge scavenging. A schematic of such a design is shown in Figure S9, in which two steams are angled (at an angle θ^o each) to aim at a common target perpendicular to their midpoint; the angle formed by the starting point of stream one, the target and stream two is 2θ. Each stream is given the same stream speed ($|\nu_{stream}| = |v_j|$) as this is a consequence of the users flow rate given identical manifolds on the inhaler.

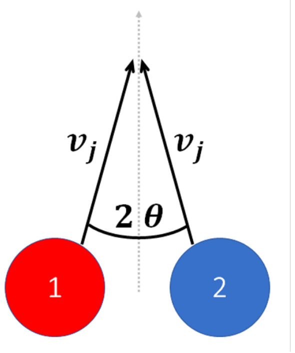

Figure S9: Definition of the angle (2θ) between stream 1 (red) and stream 2 (blue) for two streams angled equally (by θ in opposing directions) towards a common target perpendicular to their midpoint, where each stream has a velocity such that the speed of each flow is the same magnitude ($|\nu_{stream}| = |v_j|$).

References

(1) Hassan, M.; Williamson, C.; Baptiste, J.; Braun, S.; Stace, A. J.; Besley, E.; Stamm, B. Manipulating Interactions between Dielectric Particles with Electric Fields: A General Electrostatic Many-Body Framework. *Journal of Chemical Theory and Computation* **2022**, *18*, 6281–6296.

(2) Bichoutskaia, E.; Boatwright, A. L.; Khachatourian, A.; Stace, A. J. Electrostatic analysis of the interactions between charged particles of dielectric materials. *The Journal of Chemical Physics* **2010**, *133*, 24105.

(3) Baptiste, J.; Williamson, C.; Fox, J.; Stace, A. J.; Hassan, M.; Braun, S.; Stamm, B.; Mann, I.; Besley, E. The influence of surface charge on the coalescence of ice and dust particles in the mesosphere. *Atmospheric Chemistry and Physics Discussions* **2020**, *2020*, 1–16.

(4) Filippov, A. V.; Chen, X.; Harris, C.; Stace, A. J.; Besley, E. Interaction between particles with inhomogeneous surface charge distributions: Revisiting the Coulomb fission of dication molecular clusters. *The Journal of Chemical Physics* **2019**, *151*, 154113.

(5) Zheng, Z.; Leung, S. S. Y.; Gupta, R. Flow and Particle Modelling of Dry Powder Inhalers: Methodologies, Recent Development and Emerging Applications. *Pharmaceutics* **2021**, *13*.

(6) van Wachem, B.; Thalberg, K.; Remmelgas, J.; Niklasson-Björn, I. Simulation of dry powder inhalers: Combining micro-scale, meso-scale and macro-scale modeling. *AIChE Journal* **2017**, *63*, 501–516.
